# Study of the Functionalities of a Biochar Electrode Combined with a Photoelectrochemical Cell

**DOI:** 10.3390/ma16010043

**Published:** 2022-12-21

**Authors:** Spyridon Giannakopoulos, John Vakros, Ioannis D. Manariotis, Dionissios Mantzavinos, Panagiotis Lianos

**Affiliations:** 1 Department of Chemical Engineering, University of Patras, 26504 Patras, Greece; 2 School of Sciences and Engineering, University of Nicosia, Nicosia 2417, Cyprus; 3 Environmental Engineering Laboratory, Department of Civil Engineering, University of Patras, University Campus, 26504 Patras, Greece

**Keywords:** biochar, spent malt rootlets, photocatalytic fuel cell, photoelectrochemical cell, electrocatalyst, oxygen reduction

## Abstract

Biochar has been obtained by pyrolysis of spent malt rootlets under limited oxygen supply and further activated by mixing with KOH and pyrolyzed again at high temperature. The total specific surface area of such activated biochar was 1148 m^2^ g^−1^, while that of micropores was 690 m^2^ g^−1^. This biochar was used to make a functional electrode by deposition on carbon cloth and was combined with a photoelectrochemical cell. The biochar electrode functioned as a supercapacitor in combination with the electrolyte of the cell, reaching a specific capacity of 98 Fg^−1^, and it was capable of storing charges generated by the cell, proving current flow both under illumination and in the dark. The same electrode could be used as an air-cathode providing oxygen reduction functionality and thus demonstrating interesting electrocatalyst properties.

## 1. Introduction

Energy storage is a key issue in modern technology, both at low and large scales. Solar energy can now be converted to electricity at a reasonable cost; however, the intermittent nature of solar energy requires either immediate consumption or storage. In this respect, storage at a large scale is still very costly, thus prolonging the dependence on fossil fuels. On the other hand, mobile electronics have promoted the energy storage question as one of the most challenging technical issues of our times. Batteries are, of course, the best-known and straightforward means of electricity storage. Nevertheless, one other means of storing electricity gains more and more ground: supercapacitors. Supercapacitors are capacitors of very high capacity. Such capacitors are now almost exclusively made by a combination of a porous electrically conductive material with an electrolyte. When a solid conductor is charged, a Helmholtz double layer is formed at its interface with the electrolyte, and this corresponds to the accumulation of charges that can reach high levels of specific capacity (Electric Double Layer Capacitors, EDLC). Obviously, specific capacity depends on the specific electrode surface. Therefore, porosity and the specific surface is the number one parameter that may determine the specific capacity. In reality, porosity by itself is not the only crucial factor since the pores must be large enough to allow diffusion of ions through them and small enough to reach a high specific surface. These opposing factors finally determine the ideal supercapacitor, i.e., a material with the hierarchical distribution of pores involving meso, micro, and macropores, thus satisfying the above requirements. Fortunately, such materials are found in nature, and they are obtained by the carbonization of biomass. They are given the universal name biochar (BC). Biochars can be obtained from an almost unlimited number of sources. Any biomass can offer biochar. Charcoal, i.e., wood-derived biochar, is the oldest and best-known source of heat. However, biomass residues, for example, biomass wastes, can also be converted to biochar, thus transforming a potentially harmful pollutant into a useful energy source [1,2,3,4,5,6,7,8]. What is of interest in the present case is the fact that biochars are electrically conductive, as most carbonaceous materials, and since they are porous, they may and have indeed been used to make supercapacitors [1,9].

The hierarchical distribution of pores and high specific surface is not directly obtained by simple carbonization of the biomass. Carbonized biomass contains a lot of lignin and cellulose or hemicellulose, which offer nothing to conductivity and limit porosity. Such components may be removed by further treatment in a strongly alkaline environment (KOH) [5,6,7,8]. The final product is then appropriate for the construction of a supercapacitor. Nevertheless, the inherent nature of biomass of different origins may offer biochar of varying quality and components [5,6,7,8,9,10,11,12]. In the present work, we have used biochar derived from spent malt rootlets (SMR), i.e., waste created by brewery factories. This choice was made because this material has performed very well in previous studies [12,13], and it was thus considered suitable also for the present case.

**Figure 1:**
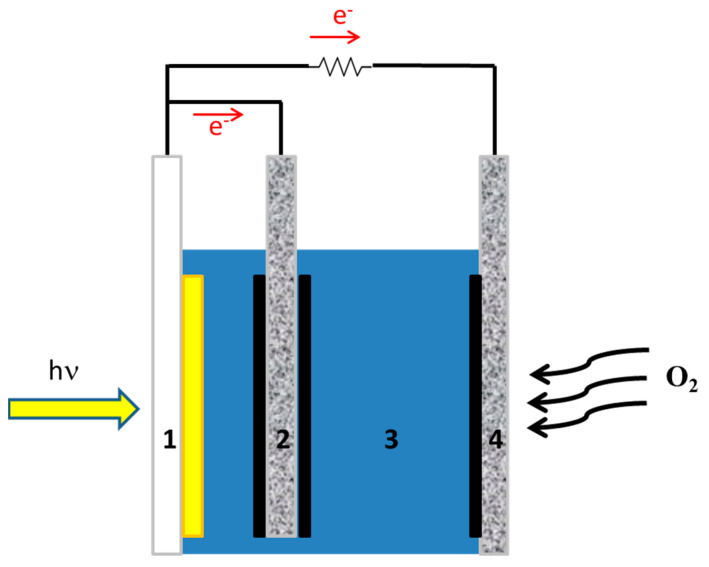
Illustration of the device with supercapacitor: (1) transparent FTO electrode carrying photocatalyst (yellow part); (2) supercapacitor electrode loaded with biochar (solid black); (3) electrolyte; (4) gas diffusion counter electrode carrying electrocatalyst (solid black).

A supercapacitor may be combined with a photovoltaic cell to store electricity. This will necessitate two separate devices, one to produce and the second to store energy. Fortunately, these two functions can be combined in one single device if, instead of a photovoltaic cell, a photoelectrochemical cell is used to convert light into electricity. A photoelectrochemical cell (usually) consists of a photoanode, a counter electrode, and an electrolyte. If a third (biochar) electrode is added to the photoelectrochemical cell, it can form a supercapacitor with the electrolyte of the cell, providing both functions of energy conversion and storage in one single device [14,15]. Such a combination is illustrated in Figure 1. The present work focuses on the functionality of such a device.

## 2. Materials and Methods

All materials were of reagent grade and were provided by Sigma-Aldrich unless otherwise specified. Thus Fluorine-doped Tin Oxide transparent electrodes (8 ohm/square) were purchased from Pilkington (Shelbyville, IN, USA), carbon cloth (CC) from Fuel Cell Earth (Wobum, MA, USA), and carbon black (CB) from Cabot Corporation (Vulcan XC72, Billerica, MA, USA).

SMR biochar was prepared from spent malt rootlets, the main byproduct of the Athenian Brewery S.A (Patras, Greece), according to a previously published procedure [13]. A weighed quantity of the dried rootlets was placed in a quartz vessel and heated at 850 °C in a gradient temperature furnace (LH 60/12, Nabertherm GmbH, Lilienthal, Germany) under a limited air supply containing only 20% of the O_2_ required to burn the biomass completely. The prepared biochar was mixed with KOH in a mass ratio KOH:Biochar = 4:3 and then pyrolyzed again at 850 °C in order to activate it. Then the collected biochar was placed in a flask with 1000 mL of triple-distilled (3D) water and heated under reflux for 1 h to a temperature slightly above 105 °C. After heating, the suspension was cooled to room temperature, filtrated under vacuum, washed with 3D water, and dried at 120 °C for 2 h. This biochar was used for the preparation of the supercapacitor electrode. 

The device employed for the present work is schematically illustrated in Figure 1. The photoanode electrode was made by successively depositing mesoporous titania (TiO_2_) on an FTO electrode, followed by sensitization with CdS nanoparticles incorporated into the titania mesoporous structure by the SILAR method (Successive Ionic Layer Adsorption and Reaction) [16,17]. The procedure was the same as the one repeatedly used in the past; for this reason, it is detailed only in the Supporting Information file. The counter electrode and the biochar electrode were made by deposition of carbonaceous materials on carbon cloth electrodes. Carbon black was deposited on carbon cloth by the following procedure. A paste was first made by the vigorous stirring of 300 mg of carbon black dispersed in 10 mL water, to which 100 mg PTFE (Polytetrafluoroethylene, 60 wt % dispersion in water) was added as a binder. Then the paste was spread on the carbon cloth with a spatula, dried at 100 °C, and finally annealed at 340 °C. This procedure was repeated to ensure full coverage of the active electrode area with carbon black. In the case of biochar deposition, the same procedure was followed, but instead of 300 mg of carbon black, a mixture of 270 mg of biochar with 30 mg of carbon black was used. The presence of 10 wt % of carbon black was necessary to make a more stable film. The active area of all three electrodes was 1 cm^2^. The total carbon loaded on the carbon cloth electrode was 15 mg. The electrolyte was 0.5 M aqueous NaOH, to which 10 v% ethanol was added. The presence of ethanol is necessary to increase cell current by acting as a scavenger of photogenerated holes. Ethanol is oxidized during cell operation, thus playing the role of fuel. In most measurements, no reference electrode was used. When needed, an Ag/AgCl electrode was used as a reference electrode. The photoanode was irradiated by using a Xenon lamp with a light intensity of approximately 100 mW cm^−2^. Current-voltage measurements were made with an Autolab potentiostat PGSTAT128N (Utrecht, The Netherlands).

The determination of the Specific Surface Area (SSA) and pore size distribution of the prepared biochar was performed with the N_2_ adsorption-desorption isotherms at liquid N_2_ temperature (Tristar 3000 porosimeter, Micromeritics Norcross, GA, USA), the point of zero charges was measured with the Potentiometric Mass titration technique (Tim Talk 8 Radiometer, Copenhagen, Denmark), while the morphology of the biochar was examined using a scanning electron microscope (SEM) (FEI Quanta 250 FEG, Hillsboro, OR, USA) working under various pressures (10–4000 Pa). X-ray diffraction (XRD) patterns were recorded with a Bruker D8 (Billerica, MA, USA) Advance diffractometer equipped with a nickel-filtered CuKa (1.5418 Å) radiation source. Fourier transform infrared (FTIR) spectroscopy was performed using a Perkin Elmer Spectrum RX FTIR system (Waltham, MA, USA). The measurement range was 4000–400 cm^−1^. More details about the characterization can be found in [12,13].

## 3. Results and Discussion

### 3.1. Basic Characterization of the Biochar

The biochar was prepared by pyrolysis at 850 °C, and it was activated with KOH. More specifically, biochar and KOH were mixed in a ratio of 4:3 (KOH:BC) and pyrolyzed again at 850 °C. This second pyrolysis caused intense changes in BC and significantly altered its physicochemical properties. Although the mechanism of the KOH action is not fully known, the following model may presently apply. KOH reacts with the carbon phase and produces carbonates and potassium. KOH melts at 360 °C while the potassium in metal form has a boiling point of about 760 °C. At the beginning, KOH is transformed to K_2_O and H_2_O, which can react with C to form CO and CO_2_ and finally K_2_CO_3_, as shown in Equations (1)–(4):

2 KOH → K_2_O + H_2_O (1)

H_2_O + C → CO + H_2_ (2)

H_2_O + CO → CO_2_ + H_2_ (3)

K_2_O + CO_2_ → K_2_CO_3_ (4)

The potassium compounds can react with C, and potassium is reduced to metallic K, as in Equations (5)–(7):

CO_2_ + C → 2 CO (5)

K_2_CO_3_+ 2 C → 2 K + 3 CO (6)

K_2_O + C → 2 K + CO (7)

Alternatively, the reaction in Equation (8) can also take place:

6 KOH + 2C → 2 K + 3 H_2_ + 2 K_2_CO_3_ (8)

Based on the above reactions, the various K compounds etch the carbon framework, generating an extended pore network, while K itself can efficiently intercalate into the carbon lattices, which results in their expansion. Even after the removal of K, either by high temperature or by washing after the pyrolysis, the expanded carbon lattices cannot return to their previous structure [18]. 

**Figure 2:**
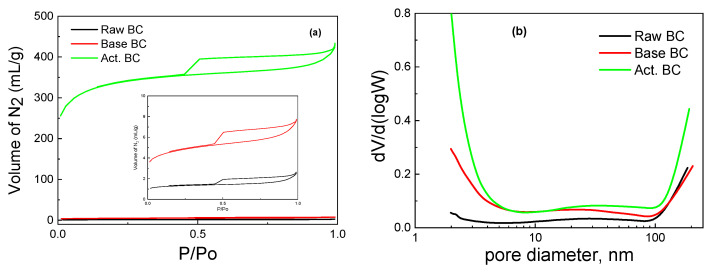
Adsorption-desorption isotherms (**a**) and Pore size distribution (**b**) for the studied biochars.

The activation of BC with KOH under pyrolysis is a process that combines high temperature with the influence of the base. Compared to BC pyrolyzed in the absence of KOH (raw BC) and BC treated with a basic solution under mild temperature (105 °C), there are significant differences in the physicochemical characteristics of the biochar. Almost all the applied physicochemical techniques exhibit these differences. This can be seen in Table 1, where the values of specific surface area (SSA), micropores SSA, pore volume, and point of zero charges are reported. The pyrolysis of the raw biomass produced biochar with a moderate SSA, a high amount of minerals, and a slightly basic surface. The surface groups were mainly oxygen-containing species, and the graphitic phase was not well detected. Treatment with a basic solution considerably increased the SSA and the surface basicity, while the activation of BC with the combined use of pyrolysis with KOH further increased the surface acidity and the SSA of the activated BC. The N_2_ adsorption-desorption isotherms of the samples are presented in Figure 2. It is seen that the amount of adsorbed N_2_ is by far higher in the activated BC. Interestingly, the type of the isotherm is not altered with any of the processes. The isotherm is typical of the IV types with an H_4_ hysteresis loop according to the IUPAC classification [19,20]. The high amount of adsorbed N_2_ in the very low p/Po values (about 250 mL g^−1^) is evidence of the high microporosity of the activated sample. Indeed, the micropores SSA was equal to 690 m^2^ g^−1^.

The pore size distribution was not significantly different in the three samples. As can be seen in Figure 2b, all three BC have micro, meso, and macropores. Nevertheless, the corresponding micropore SSA values broadly changed in going from raw BC to KOH-activated BC, as expected, owing to the removal of carbon species from the biochar (mainly lignin and cellulose, as already said). The above equations describe the routes through which such removal is made possible. 

**Figure 3:**
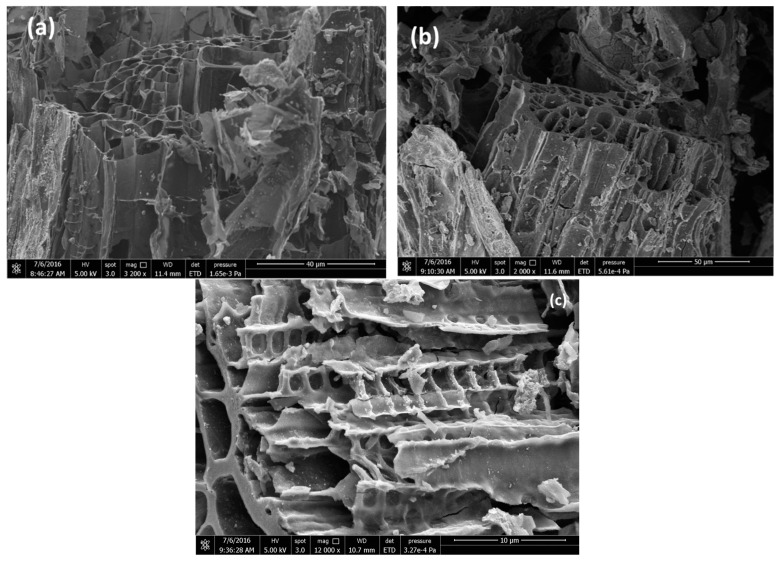
SEM images of the biochars studied; (**a**) Raw BC, (**b**) Base-treated BC, (**c**) Activated BC.

The pore structure in the studied BC can be seen in the SEM images of Figure 3, while the corresponding EDX analysis is given in Table 2.

As can be seen from Table 2, the BCs consist mainly of C and O (more than 94%). The activated BC had, even more C and O, exceeding 98%. The K content in activated BC is low as a result of the high pyrolysis temperature above the melting point of K. 

**Figure 4:**
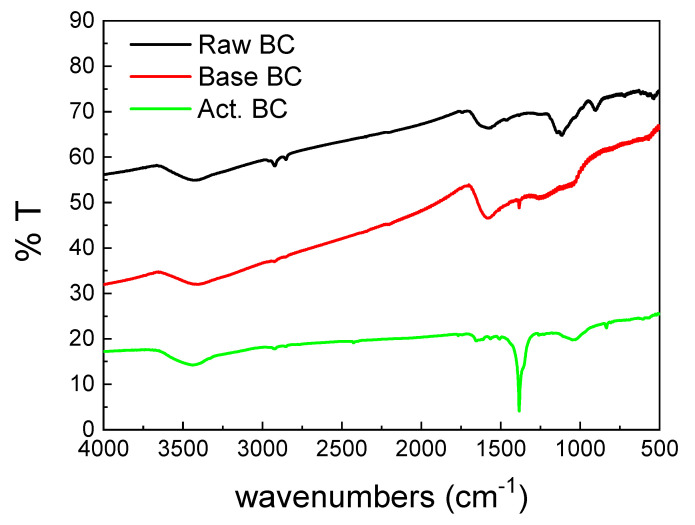
FTIR spectra of the studied biochars.

The FTIR spectra of the three BC samples (Figure 4) were also different. The main peak of the activated BC (Act.BC) is centered at 1383 cm^−1^ and corresponds to the carbonate species. This is expected as the result of the reactions presented in Equations (4) and (8). The formation of carbonates alters the spectrum of the Act. BC, while the other two spectra have similarities. This points out that pyrolysis is an important parameter for the differences observed between the three biochars. The carbonate species are absent in the raw BC, and only a small amount can be detected in the base-treated (base BC). This is probably due to the oxidation of the oxygen-containing groups during treatment. All the samples had a broad peak centered at about 3400 cm^−1,^ which is due to the surface–OH groups or absorbed water molecules [21]. The double peak below 3000 cm^−1^ is higher in the raw BC. This peak is characteristic of the aliphatic H. This H is removed in the second treatment, and it is evidence that the treatment increases the graphitization of the BC. 

**Figure 5:**
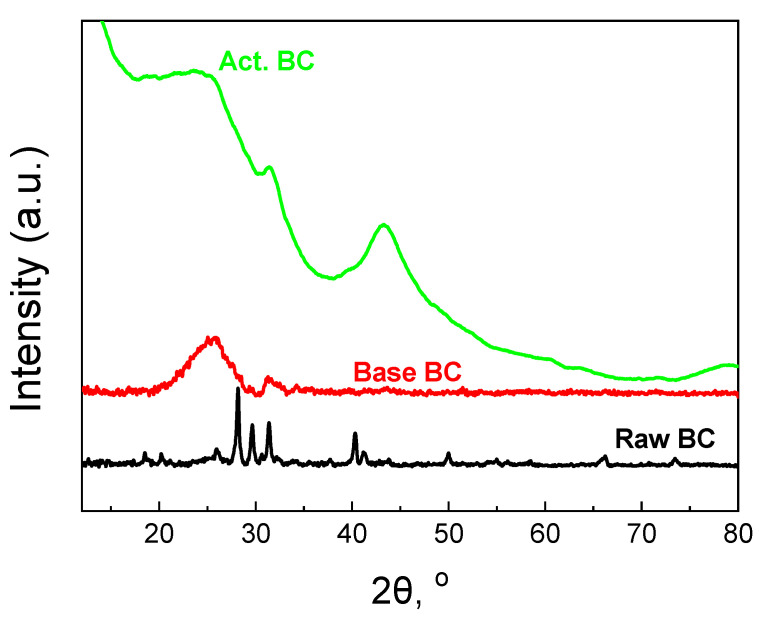
XRD patterns of the studied biochars.

The XRD patterns of the BC are also informative. The patterns are presented in Figure 5. Raw BC exhibits many sharp peaks due to the high amount of minerals. The content of minerals was found to be 32% and consists mainly of Mg_3_(PO_4_)_2_, Ca_3_(PO_4_)_2,_ and KCl [12,22]. During the treatment with a base solution at 105 °C, the majority of the minerals were removed from the biochar, and the amorphous graphitic phase could be then seen.

The broad peak centered at 2θ = 26° corresponds to the (002) plane of the graphitic carbon [23,24]. The peak at about 2θ = 43° for the (100) plane cannot be observed. This peak is due to the sp^2^ hybridization of the carbon phase [12,25]. The activation procedure helps the biochar to better form these two peaks. Especially, the second peak at 2θ = 43.5° for the (100) plane is well formed, the first evidence for the formation of extended carbon species with sp^2^ hybridization. Since this peak requires high pyrolysis temperature [26], the second pyrolysis step is responsible for the formation. Finally, the small peak at about 31.5°, overlapped with the broad peak of the graphitic carbon phase, can be attributed to carbonates.

**Figure 6:**
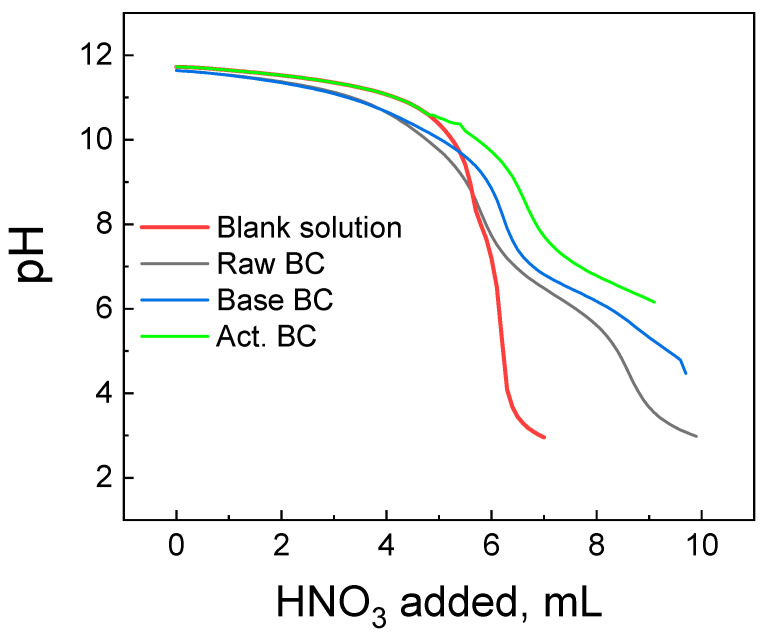
Potentiometric titration curves of the biochar’s studied and the corresponding blank solution.

The titration curves of each BC studied are presented in Figure 6, with the corresponding titration curve of the blank solution. The blank solution is the solution of the indifferent electrolyte used without the addition of the BC. The raw BC and the Base Bc titration curves have a common section point with the curve of the blank solution at pH = 8.4 (Raw BC) and 9.6 (Base BC). This pH corresponds to the pzc of each material. The picture is slightly different for the activated BC. Its titration curve and the curve of the blank solution approach each other, and the section point is not clearly seen. This is due to the high basicity of the act. BC and its pzc are >10.5 of any, above the detection limit of the Potentiometric Mass Titration technique [27].

Since the activated BC demonstrated the best characteristics, it was exclusively used to make supercapacitor electrodes which were employed for the photoelectrochemical studies that are reported in the next subsection.

### 3.2. Photoelectrochemical Studies

**Figure 7:**
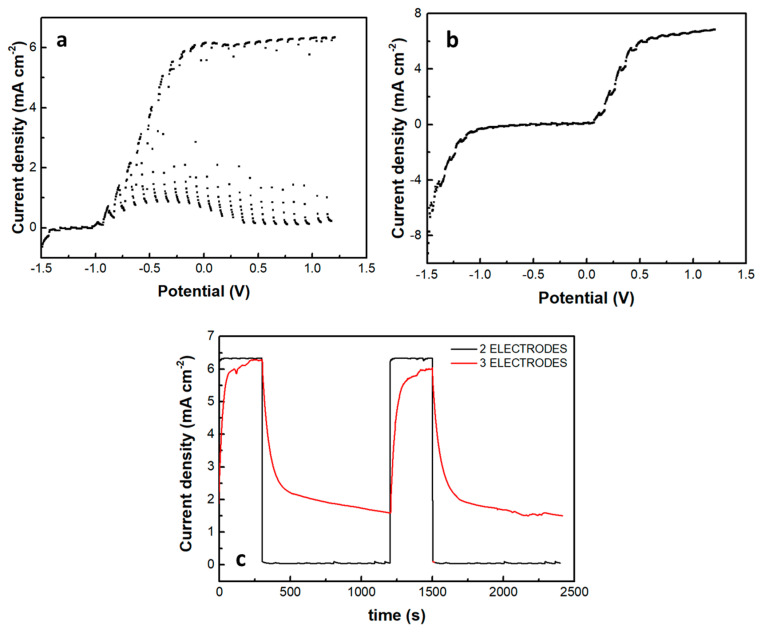
Current density-voltage curves recorded in the light chopping mode to demonstrate the conditions for photocurrent production: (**a**) recorded with a 2-electrode cell comprising a photoanode and a cathode electrode and (**b**) recorded with a 3-electrode cell comprising photoanode, cathode and supercapacitor electrode. Chopping had a period of 20 s, i.e., 10 s illumination and 10s dark. In (**a**), the current drops fast in the dark, but in (**b**), the supercapacitor electrode does not allow the current to decrease. (**c**) Shows the behavior of both systems in a longer chopping period, i.e., 5 min light and 15 min dark, under potentiostaic conditions of 0.4 V. The electrolyte was 0.5 M aqueous NaOH containing 10% w ethanol.

A photoelectrochemical cell has been made, and its current-voltage characteristics were first studied in the absence of the supercapacitor electrode. The cell consisted of a photoanode, a CB/CC cathode (i.e., a carbon cloth loaded with carbon black), and an aqueous NaOH electrolyte containing 10 v% EtOH. Figure 7a shows a linear sweep voltammetry curve recorded in the light chopping mode to reveal the conditions of photocurrent production. Indeed, photocurrent was produced in the voltage range above −1 V. The short-circuit current density was a bit larger than 6 mA cm^−2^. This is normal behavior for a photoelectrochemical cell functioning with a CdS/TiO_2_ photoanode [20]. The fact that it produces current without bias makes it a Photocatalytic Fuel Cell (PFC) with the above characteristics. In the voltage range approximately between −1 and +0.2 V, there is a dark current which is due to the adsorption of cations into the titania mesoporous structure, i.e., it is a capacitance current which is always observed with a photoanode based on mesoporous TiO_2_ [28]. Having thus defined the conditions for photocurrent production, we introduced the third (supercapacitor) electrode, short-circuited with the anode electrode and immersed in the same electrolyte, as illustrated in Figure 1. The current-voltage characteristics of this modified cell are shown in Figure 7b, again recorded in the light chopping mode. The aspect of the corresponding curve was now very different. Each chopping period lasted 20 s, i.e., 10 s light and 10 s dark. However, the current now did not go to zero in the dark, obviously, because the supercapacitor was charged and preserved current during the dark. Indeed, if the chopping period is made longer, as in Figure 7c, the current decay can be observed in the dark. In the absence of a supercapacitor, when the light was turned off, the current became fast zero. In the presence of the supercapacitor, as soon as the light was turned off, the current decayed and never became zero in the measurement range of 15 min. obviously, this combination of the three electrodes, photoanode, cathode, and supercapacitor can convert light energy into electricity and store it by exploiting its supercapacitor functionality.

**Figure 8:**
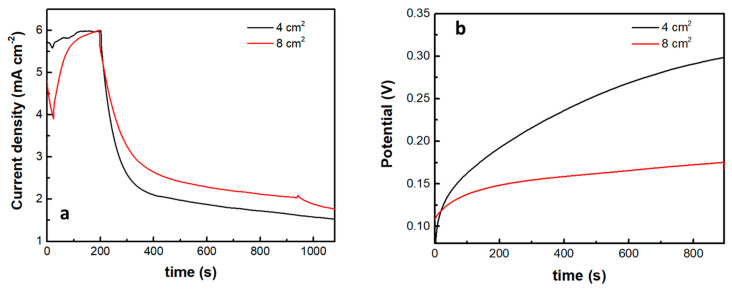
Current or voltage variation in the cell when light is turned off: (**a**) potentiostatic current drop at 0.4 V and (**b**) galvanostatic voltage increase at a current density of 1 mA cm^−2^. The insert gives the capacitor geometric area in each case.

The introduction of the supercapacitor electrode and its short-circuiting with the photoanode had a rather dramatic effect on photocurrent onset, as seen in Figure 7b. Practically this means a reduction of the photocurrent flowing in the external circuit, which is expected since the charge carriers are consumed to charge the supercapacitor. Of course, the supercapacitor charging rate will depend on the ratio between the photoanode photocurrent generation capability and the capacitance of the supercapacitor. The specific capacitance of the electrodes made by the presently activated biochar was 98 F g^−1^. Since the quantity of BC was 15 mg for an electrode of the active area of 1 cm^2^, the capacitance of such electrode was 1.47 F. Figure 8 shows discharging and charging processes for two supercapacitor electrodes, one of 4 cm^2^ and the second of 8 cm^2^ active area, obviously, possessing a capacitance of 4 × 1.47 = 5.88 F and 8 × 1.47 = 11.76 F, respectively. As expected, it takes more time to discharge and much more time to charge a larger capacitor under the same conditions. Therefore, the efficiency of charging or discharging conditions in the above cell will be a function of the relative photoanode and supercapacitor capabilities. 

**Figure 9:**
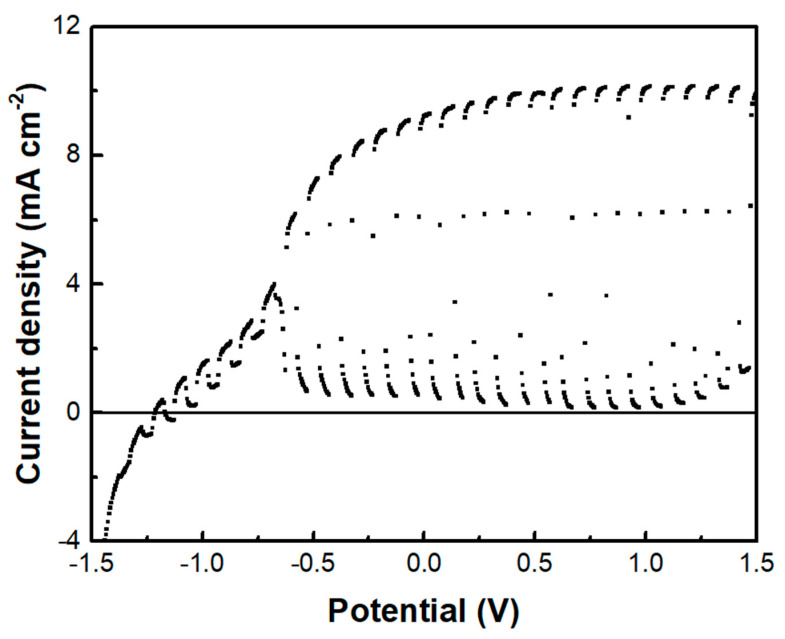
The current density-voltage curve was recorded in the light chopping mode using the BC electrode as a cathode.

**Figure 10:**
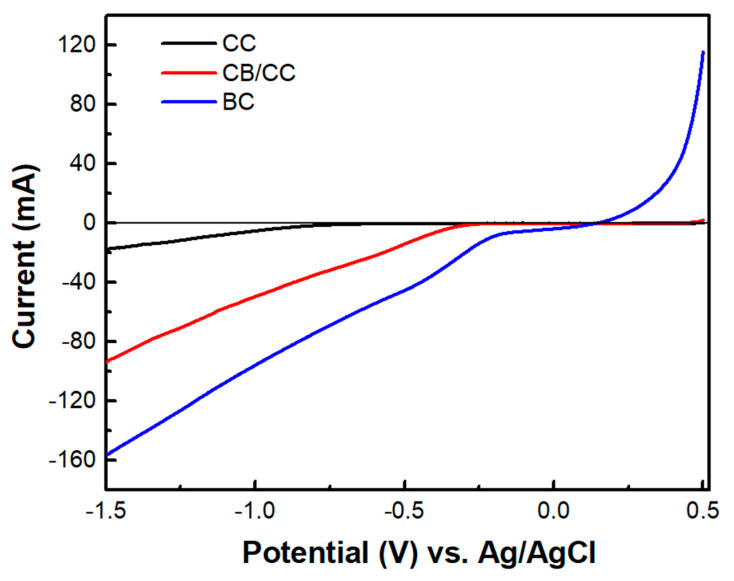
Linear sweep voltammetry curves were recorded with three different samples as working electrodes: CC carbon cloth, CB/CC carbon black on carbon cloth, and BC biochar on carbon cloth. A Pt wire was used as a counter and Ag/AgCl as a reference electrode. The electrolyte was 0.5 M aqueous NaOH.

The above data describe the behavior of the biochar electrode as a supercapacitor. This property was demonstrated, as already said, by short-circuiting the biochar electrode with the photoanode electrode so that the photogenerated charge carriers may be used to charge the supercapacitor. However, another configuration has also been examined. That is, the BC electrode was used as a cathode electrode in a two-electrode cell, i.e., comprising only a photoanode and a biochar electrode. Interestingly, in that case, there was no charge-storing functionality observed. As seen in Figure 9, the current-voltage characteristics of the cell were similar to those of Figure 7a, where the cathode electrode was a CB/CC (pure carbon black on carbon cloth) electrode. Nevertheless, the maximum current was substantially larger in the case of the biochar electrode. Such a CB/CC electrode has a relatively negligible capacitance, being far below 1 F g^−1^. Obviously, when the BC electrode is used as the cathode, BC functions only as an oxygen reduction catalyst. In order to compare the oxygen reduction functionality between the BC and the CB/CC electrode, current-voltage curves have been recorded, as in Figure 10, by employing the two electrodes as working electrodes. A Pt wire was then used as the counter electrode and Ag/AgCl as a reference electrode. The reductive capacity of both electrodes is then demonstrated; however, the BC electrode was activated at a lower negative bias, indicating superiority against the CB/CC electrode. This result is interesting since it shows that a material originating from a waste, i.e., from spent malt rootlets, may function as a satisfactory electrocatalyst to operate a photoelectrochemical cell. Figure 10 also shows, as expected, that carbon cloth alone had poor electrocatalytic capacities. 

## 4. Conclusions

Activation with KOH is necessary in order to obtain biochar with a substantial specific surface area. Such biochar can be used to construct a supercapacitor electrode and introduce it in a photoelectrochemical cell providing both energy conversion and energy storage functionality. The biochar acts as a supercapacitor in combination with the electrolyte of the photoelectrochemical cell. Photogenerated electrons are directly stored in the supercapacitor electrode; however, if the biochar electrode is used as an air-cathode, it acts as an oxygen reduction catalyst demonstrating a higher reduction capacity than standard nanoparticulate carbon electrocatalyst (like carbon black). Therefore, activated biochar’s may prove themselves as useful electrocatalysts.

## Figures and Tables

**Table 1:** Characterization data of the employed biochar.

Sample	Preparation Method	SSA (m^2^ g^−1^)	Micropores SSA (m^2^ g^−1^)	Pore Volume (mL g^−1^)	Point of Zero Charge
Raw BC	Pyrolysis or raw biomass at 850 °C	100	58	0.077	8.4
Base-treated low temp. BC	Treatment of raw BC at 105 °C with 1M NaOH	363	175	0.247	9.6
Activated BC	Pyrolysis of raw BC at 850 °C mixed with KOH	1148	690	0.99	>10.5 if any

**Table 2:** % atomic concentration of the different elements of biochars studied.

Element	% Atomic Concentration		
	Raw BC	Base Treated BC	Activated BC
C	84.2	83.2	88.0
O	10.3	11.1	10.2
Na	N.D.	1.7	0.7
K	2.4	0.7	0.3
Mg	0.2	0.6	N.D.
Si	1.0	0.3	N.D.
P	1.0	1.4	N.D.
S	0.2	0.2	0.8
Cl	0.5	0.1	N.D.
Ca	0.2	0.7	N.D.

N.D.: not detected.

## Data Availability

Available on reasonable request.

## Supplementary Materials

The following supporting information can be downloaded at: https://www.mdpi.com/article/10.3390/ma16010043/s1, Construction of the photoanode electrode. Reference [29] are cited in the supplementary materials.
