# Supplementary material for: Study of the Functionalities of a Biochar Electrode Combined with a Photoelectrochemical Cell
Source: Materials (Basel). 2022 Dec 21;16(1):43. doi: 10.3390/ma16010043 (PMC9821603; doi:10.3390/ma16010043)
Supplement: Supplementary file 1 [file materials-16-00043-s001.zip › materials-2083862-supplementary.pdf]

materials

Supporting information

Study of the Functionalities of a Biochar Electrode Combined with a Photoelectrochemical Cell

Spyridon Giannakopoulos[1], John Vakros*[1,2], Ioannis D. Manariotis[3], Dionissios Mantzavinos[1] and Panagiotis Lianos *[1]

Department of Chemical Engineering, University of Patras, 26504 Patras, Greece spyrosgiannak@gmail.com; vakros@chemistry.upatras.gr; mantzavinos@chemeng.upatras.gr; lianos@upatras.gr

[2] School of Sciences and Engineering, University of Nicosia, 2417 Nicosia, Cyprus vakros@chemistry.upatras.gr

[3]Department of Civil Engineering, Environmental Engineering Laboratory, University of Patras, University Campus, GR-26504 Patras, Greece idman@upatras.gr

*Correspondence: vakros@chemistry.upatras.gr (JV); lianos@upatras.gr (PL)

Construction of the photoanode electrode

An FTO glass was cut in the required dimensions. It was then cleaned thoroughly with soap. Further cleaning was achieved by sonicating the glass in acetone, ethanol and water. A TiO_2 precursor solution was prepared by mixing 3.5 g of Triton X-100, 19 mL of ethanol, 3.4 mL of glacial acetic acid and 1.8 mL of titanium isopropoxide. A layer of titania was deposited by dipping the clean FTO in the precursor solution. The deposition was limited within the required electrode surface with the help of tapes to avoid coating the back of the FTO glass. After removing the tapes, the electrodes were air dried before calcinating at 550°C. The same procedure was repeated for a second time to ensure a uniform layer. Then, a mesoporous titania layer was deposited on the electrode by doctor blading a paste based on P25 Degussa powder. The paste was prepared as explained in previous publications [1,2]. To obtain an approximate thickness of 10μM the process was repeated for the second time. This layer was also calcined at 550°C. CdS sensitization was done by Successive Ionic Layer Adsorption and Reaction (SILAR). 0.1M cadmium nitrate and 0.1M of sodium sulfide were used as the source of cadmium and sulfide ions, respectively. The SILAR cycles were repeated until a bright yellow color was observed on the electrode surface (approximately 10 cycles). The electrode was first dried under the flow of nitrogen and then in an oven at 70°C.

References

1. Ito, S., Chen P., Comte, P., Nazeeruddin, M. K., Liska, P., Pechy P. and Gratzel M., Fabrication of Screen-Printing Pastes From TiO2 Powders for Dye-Sensitised Solar Cells. *Prog. Photovolt: Res. Appl.* 2007, 15, 603-612 https://doi.org/10.1002/pip.768
2. Sfaelou, S., Sygellou, L., Dracopoulos, V., Travlos, A., Lianos, P., Effect of the Nature of Cadmium Salts on the Effectiveness of CdS SILAR Deposition and Its Consequences on the Performance of Sensitized Solar Cells. *J. Phys. Chem. C* 2014, 118, 22873–22880 https://doi.org/10.1021/jp505787z
